# Comparing quantitative and qualitative verbal and social autopsy tools: does a qualitative supplement improve understanding of the social determinants of under-five deaths in the slums of Kampala, Uganda?

**DOI:** 10.29392/001c.38743

**Published:** 2022-10-22

**Authors:** Amy W Blasini, Peter Waiswa, Ann Wolski, Phillip Wanduru, Chelsea Finkbeiner, Ashura Bakari, Lucky Amutuhaire, Cheryl A Moyer

**Affiliations:** 1 University of Michigan Medical School,; 2 Health Policy, Planning, and Management, Makerere University School of Public Health; Global Public Health, Karolinska Institute,; 3 Ghana Health Service,; 4 Population Studies, Makerere University,; 5 Learning Health Sciences and Obstetrics & Gynecology, University of Michigan Medical School

**Keywords:** U5 mortality, social autopsy, qualitative methods

## Abstract

**Background:**

Understanding biological causes of death and sociocultural factors influencing outcomes is critical to reducing mortality in low-resource settings. Verbal and Social Autopsy instruments (VASAs) query family members about events leading to an individual’s death, resulting in quantitative, categorical data. This study sought to determine the value of a supplemental in-depth qualitative interview (VASA-QUAL).

**Methods:**

This cross-sectional study was conducted in two slum neighborhoods in Kampala, Uganda, among families who lost a child under five within the preceding six months. A trained, local researcher conducted the quantitative VASA and then administered the VASA-QUAL to family members. Quantitative data were analyzed using Stata V16.0; qualitative data were transcribed into English and analyzed using NVivo V12.0. The biomedical cause of death was determined using a panel of physicians to code verbal autopsy items. Quantitative VASA variables were compared with qualitative variables from the VASA-QUAL using a rubric of indicators derived from the Pathways to Survival framework. Kappa statistics and percent agreement were calculated to compare quantitative and qualitative data. Three coders independently rated whether qualitative data provided additional information that improved understanding of the cause of death.

**Results:**

48 VASAs were conducted (child age range: 1 month to 52 months). Agreement on key indicators ranged from 81.2% (place of death) to 93.8% (recognition of illness), with Kappa coefficients ranging from −0.038 to 0.368. The qualitative component added or clarified information about pediatric illness and care-seeking across all indicators, including recognition of illness (94.0%), care-seeking decisions (79.0%), whether home care was provided (73.0%), and choice of outside care (85.0%). Qualitative interviews frequently included symptoms missing or denied in the quantitative VASA and clarified the chronological order of symptoms. Many qualitative interviews described complicated mechanisms of decision-making not captured in the quantitative survey. Both agreement across data types and whether meaningful information was added by the qualitative data varied by cause of death, although our sample size limited our ability to conduct statistical analysis in this regard.

**Conclusions:**

Supplementing quantitative VASA tools with an in-depth VASA-QUAL interview provided important additional information, but not consistently across indicators or causes of death. Despite challenges associated with feasibility, supplemental qualitative interviews may be an important tool for understanding the complexity of events leading up to childhood deaths.

Approximately 5 million children under the age of 5 (U5) die each year, representing an enormous improvement over the more than 12 million children U5 that died in the early 1990s.^1^ Nonetheless, children in sub-Saharan Africa have a disproportionate risk of U5 death, with rates 14 times higher than children in Europe and North America.^1^ Infectious diseases, including pneumonia, diarrhoea and malaria, and nutrition-related illnesses, are often listed as leading causes of death, after the neonatal causes that account for nearly half of U5 deaths.^1^ Infectious and nutrition-related illnesses are preventable and treatable, suggesting unacceptable rates of avoidable mortality among African children.

In settings with limited vital registration systems, identifying and documenting biomedical causes of death among children is often achieved through the use of a technique known as verbal autopsy (VA). VA is the process of interviewing close contacts of the person who died to identify probable causes of death, typically comprising structured, closed-ended questions about specific signs and symptoms, e.g., ‘in the illness preceding death, did the deceased have a fever?’.^2^ Most VAs include an open narrative section in which the interviewer asks the respondent to summarize in their own words what happened in the time leading up to death. Yet, whether this is recorded verbatim, summarized as a list of key events, or enhanced with interviewer prompts varies widely. The quality of these open narratives also varies by the interviewer’s skill and training.^2^

Researchers have expanded the traditional VA methodology to include additional questions related to the social and cultural factors that may impact outcomes, to understand factors associated with avoidable mortality.^3^ Thus, “social autopsy” combines with a verbal autopsy to yield the VASA methodology – Verbal And Social Autopsy. VASA tools mirror VA tools in their emphasis on quantitative questions, with an unstructured ‘open narrative’ as a supplement to understanding the event of death. Some authors have criticized open narratives as being too long or including too much extraneous information to be useful.^4^

As members of the World Health Organization Social Autopsy Working Group and as technical consultants on the Countrywide Mortality Surveillance for Action (COMSA) project based at Johns Hopkins University, two of the authors (CAM, PW) worked to develop supplemental qualitative VASA modules that could be used to understand better the ‘how’ and ‘why’ of U5 deaths. These modules included specific open-ended questions and prompts, in keeping with qualitative data collection methodology, and required interviewers to record and transcribe the interactions verbatim. Modules focused on areas in which VASA quantitative data had not always been consistent (e.g. “I have no concerns keeping me from seeking care for illness” and then not seeking care for illness^5^) or on areas that were difficult to assess using closed-ended questions.

Given the labour-intensive nature of qualitative data collection and analysis, it is important to understand the added value of qualitative data, and whether there are specific scenarios in which it might be especially valuable. Collecting supplemental in-depth qualitative data for all cases identified during a nationwide, population-level VASA study would be impractical. Yet qualitative data guided by more than a simple open narrative prompt may be extremely helpful for smaller studies or those with specific areas of interest.

This study sought to use U5 deaths in two neighbourhoods in Kampala, Uganda, over a six-month period to compare quantitative VASA survey questions with the qualitative data from the supplemental qualitative modules. We sought to first understand the agreement between quantitative and qualitative data, not as a validation of either tool since there is no gold standard for Social Autopsy, but instead to understand if the different types of data were uncovering contradictions in family members’ reports. Then we sought to determine whether qualitative data added to our understanding of the cause of death and whether these factors varied by cause.

## METHODS

This cross-sectional study in Kampala, Uganda, utilized quantitative Verbal and Social Autopsy methodology and an in-depth qualitative supplement to compare the identified contributors and causes of U5 deaths across quantitative versus qualitative methodologies.

### STUDY SETTING

This study occurred in Kampala, Uganda, with data collection between February 6 and March 15, 2020. Specifically, the study was conducted in two parishes in the Rubaga division, one of the capital city’s five divisions. Overall, Kampala has an estimated population of 1.5 million people,^6^ more than two-thirds of whom reside in informal settlements.^7^ This study focused on the Kawaala and Nakulabye parish in the Rubaga division. Within Kawaala parish are ten geographic zones and an approximate total slum population of 92,000 people.^8^ Within Nakulabye parish are nine geographic zones and an approximate slum population of 40,000 people.^8^ Kawaala and Nakulabye parish were purposively selected due to their proximity to a public health facility, Kawaala Health Center IV. Their proximity to a government-sponsored facility helps mitigate factors like cost and transportation when evaluating the care-seeking behaviors of residents. Village Health Team members, known locally as “VHTs,” serve as voluntary community health workers and act as a bridge between the slum communities and the health center(s)/health system.

### STUDY PARTICIPANTS

Study participants were adult caregivers who lost a child aged 1 month to 60 months (5 years old) in the six months prior to data collection. Families needed to be living in the slum communities of Kawaala and Nakulabye at the time of the child’s death, and respondents needed to be over the age of 18, able to communicate in either English or Luganda, the local language, and knowledgeable about the events leading up to the death. Participants were identified through 20 VHTs working in the two neighborhoods. The VHTs sought to identify every family who had lost a child within the study window, and while there is no way to ensure that was accomplished, the VHTs felt certain they had exhausted the potential subject pool. Two families approached declined to participate without giving a reason, and one family was out of town during the interview period.

### ASSESSMENT TOOLS

This study utilized the INDEPTH Network’s Verbal and Social Autopsy tool,^9^ and a supplemental qualitative module developed in partnership with the Institute for International Programs at Johns Hopkins University, known as the VASA-QUAL.

The INDEPTH Network Verbal And Social Autopsy (VASA) questionnaire for children under the age of 5 blends the Population Health Metrics Research Consortium (PHMRC) verbal autopsy questionnaire to determine the biomedical cause of death with a modified social autopsy questionnaire to understand the illness events leading up to the death.^10^ The VASA-QUAL is a series of open-ended, semi-structured questions focusing on illness recognition, care-seeking, pregnancy and birth-related norms, and referrals. The VASA-QUAL is conducted in addition to the ‘open narrative’ portion of the VASA that asks respondents to describe what happened in their own words.

### DATA COLLECTION

Families identified by local VHTs as potentially eligible were approached by the VHT and asked about their willingness to learn more about the study. Those who indicated willingness were introduced to a trained, local research team member (bilingual in English and Luganda, the local language), who described the study, verified the participant’s eligibility and took the participant through a written informed consent process. For those with literacy challenges, the consent form was read aloud. The researcher then conducted the quantitative VASA, followed by the in-depth VASA-QUAL. Interviews were conducted in Luganda, and data collection typically lasted 60–90 minutes. Hard copies of each hand-completed survey were scanned into .pdf format, and then quantitative data were entered into a Microsoft Excel (Redmond, WA) spreadsheet. The audio-recorded portion of the interviews were transcribed from Luganda into English, leaving intact any words or phrases that were particularly difficult to translate. The study team reviewed all transcripts for accuracy and completeness, which involved the lead researcher (AB) and the interviewer (LA) reviewing and discussing the transcript.

### DATA ANALYSIS

Quantitative data were entered into Excel (Microsoft, Inc., Redmond, WA, USA) and then imported into Stata V.15.0 (StataCorp, College Station, TX, USA) to calculate summary statistics. Qualitative data were entered into NVivo 12.0 (QSR International, Melbourne, Australia) for organizational purposes.

The Pathways to Survival Framework^11^ was used to generate eight domains to compare the quantitative and qualitative findings. The eight domains include recognition of illness, care-seeking (any), use of home care, type of outside care sought, place of death, referrals for those who left the facility alive, whether followed referral for those referred, and whether followed home instructions for those discharged without a referral. Given the small number of families in our sample for whom referrals were given, we limited our detailed analysis to the five domains of recognition of illness, care-seeking (any), use of home care, type of outside care sought, and place of death. At least two coders (AB, AW, or CF) reviewed each .pdf of the quantitative survey, followed by the corresponding qualitative supplement. Then each coder indicated whether the quantitative and qualitative data agreed (0 = no, 1 = yes), and then if the qualitative supplement added anything ‘meaningful’ to the quantitative data that improved understanding of the cause of death (0 = no, 1 = somewhat, 2 = a great deal). ‘Meaningful’ was defined as something that might change how a symptom, disease presentation, event, or action was construed to understand the events leading up to death compared to the quantitative data. For example, if the quantitative data indicated care-seeking at the local health facility (yes/no), and the qualitative data included a discussion of how the family was sent home the first time they went with symptoms only to return the next day, it would be determined that the qualitative data added ‘meaningful’ information. Coding was conducted separately and then compared. When the two coders did not agree, a third coder (CAM) reviewed the data separately and served as the tiebreaker. Percent agreement and Kappa statistics were calculated regarding whether the quantitative and qualitative data agreed. Significance was set at P<0.05.

Causes of death were determined by three separate pediatric physician coders (one from Uganda, two from Ghana) trained in the Verbal Autopsy methodology. Two physicians conducted initial coding, with a third serving as the tiebreaker in case of disagreement between the first two coders, as is standard VA practice.^12,13^

### ETHICS

This study was reviewed and approved by the Makerere University School of Public Health Research and Ethics Committee, the University of Michigan Institutional Review Board, and the Uganda National Council for Science and Technology.

## RESULTS

We identified 48 children under five who had died within the previous six months in these two slum neighborhoods. Table 1 illustrates the sample demographics, including that they were equally divided across male and female children, more than half died before their first birthday, and nearly a third died at home. More than 8 in 10 children underwent some treatment at home, and the same percentage sought treatment outside the home. Eighty-three percent (83.3%) reported seeking care outside the home, while 79.2% reported receiving treatment.

**Figure 1.**
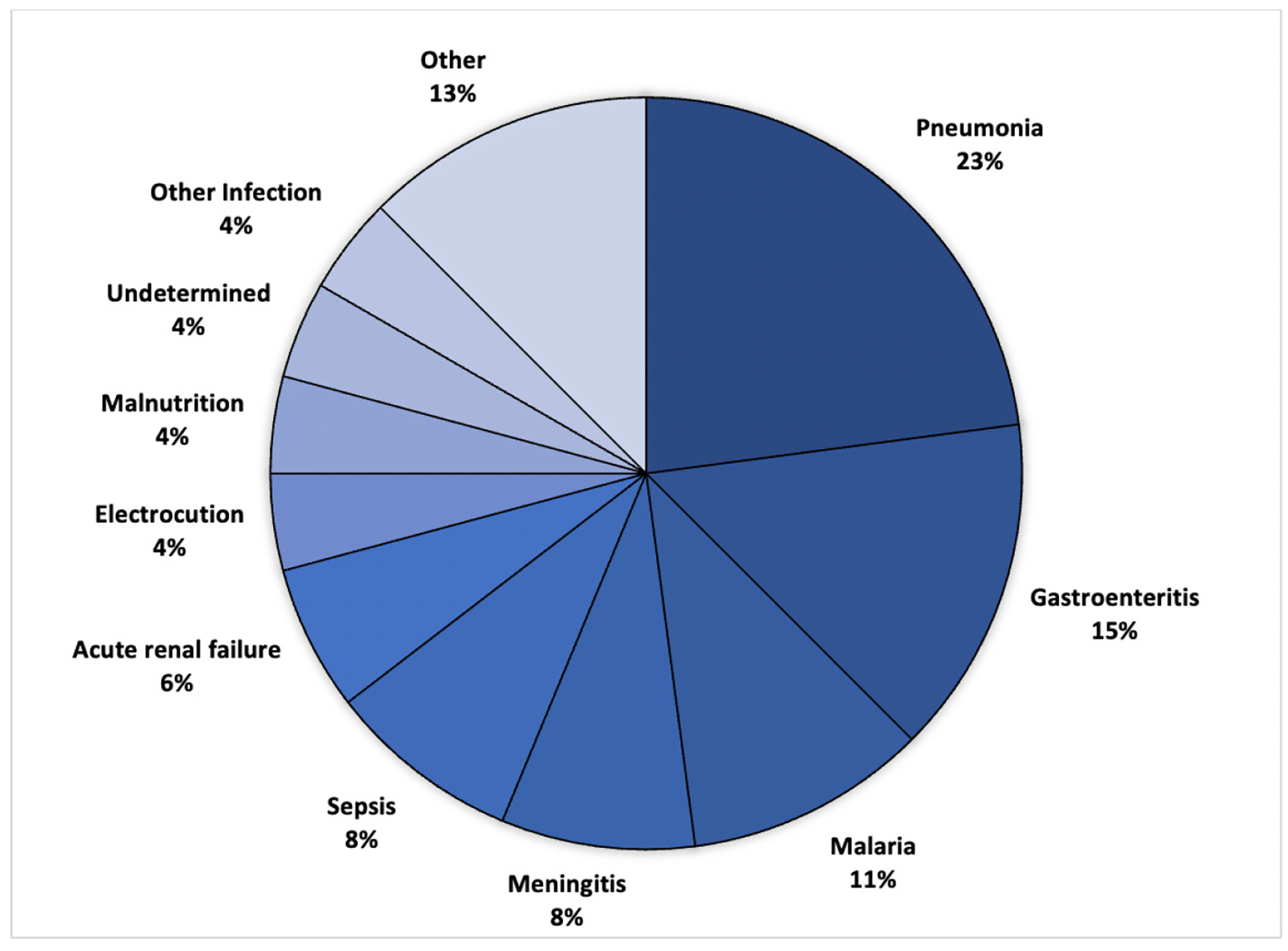
Clinical causes of death as determined by Verbal Autopsy

The leading clinical causes of death were determined to be pneumonia (N=11, 24%), gastroenteritis (N=7, 15%), malaria (N=5, 11%), meningitis (N=4, 9%), and sepsis (N=4, 9%). The remaining categories of causes of death included acute renal failure (N=3), electrocution (N=2), malnutrition (N=2), other infections (N=2), undetermined (N=2), prematurity (N=1), intussusception (N=1), severe hemolytic anemia (N=1), encephalitis (N=1), SIDS (N=1), congenital heart disease (N=1). Figure 1 illustrates the clinical causes of death.

When comparing the quantitative data with qualitative data across the five domains, more than 80% of the time, the quantitative data agreed with the qualitative data. (See Table 2.) The domain with the least amount of agreement was the place of death (81.25%, Kappa −0.038, p=0.674). Disagreements regarding the place of death often related to whether the child died on the way to the facility or died at the facility.

**Figure 2.**
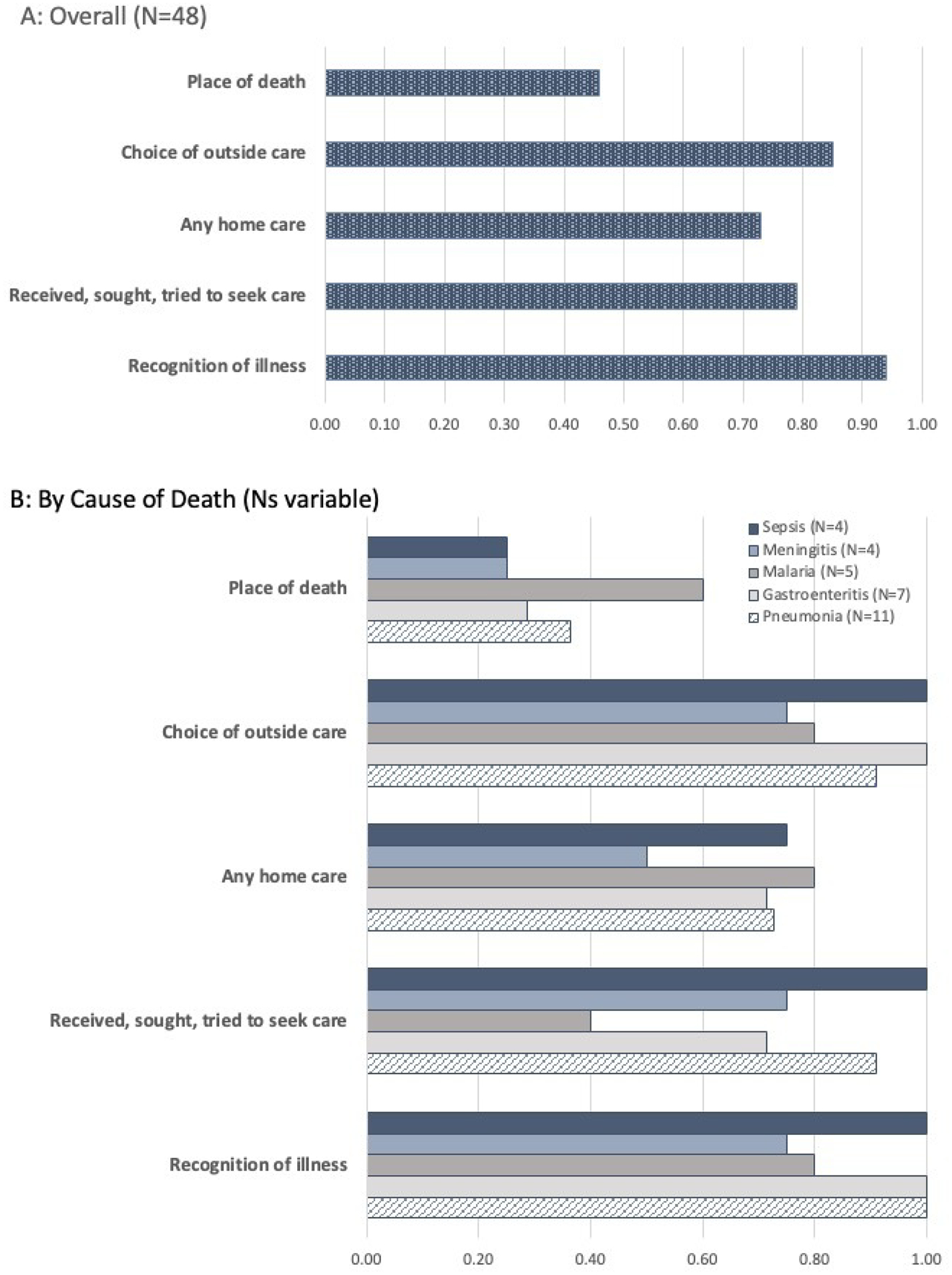
Percent of interviews in which the qualitative supplement added meaningful information to improve understanding of cause of death (Panel A: Overall (N=48); Panel B: By cause of death (Ns variable)

Figure 2 illustrates the percentage of cases in which the qualitative supplement added meaningful information to the standard quantitative VASA to help understand the cause of death. Overall, place of death was the domain least likely to be informed by the qualitative supplement (46%). Recognition of symptoms of illness was the domain most likely to be informed by the qualitative supplement (94%), followed by choice of care sought outside the home (85%). (See Figure 2A.) Figure 2B illustrates the percentage of cases in which the qualitative supplement added meaningful information, stratified by the leading causes of death. Note that the Ns for each cause of death are small, yet a place of death remained the least likely to benefit from a qualitative supplement, and both recognition of illness and choice of outside care were more likely to benefit from additional qualitative information. Examples of added information included such things as symptoms not recorded in the quantitative tool but described in the qualitative interview, detailed qualitative descriptions of how symptoms presented over time and may have stopped and started, or descriptions of multiple trips to the same facility for the same illness that was not captured in the quantitative tool. Table 3 describes additions from the qualitative supplement that were considered meaningful for each domain.

## DISCUSSION

In this comparison of quantitative and qualitative VASA data regarding U5 deaths in two slum neighborhoods in Kampala, Uganda, more than 80% of the time, there was agreement across the two different types of data. Twenty percent of the time, however, data were not in agreement. Qualitative data were most likely to add significant information regarding recognition of symptoms and choice of care sought outside the home, and deaths from pneumonia, gastroenteritis and sepsis were most likely to benefit from additional qualitative data.

Our findings align with previous research that explored the value of the open narrative portion of verbal autopsies and VASAs. In one study of interrater reliability among verbal autopsy interviewers, King et al.^2^ documented the value of the ‘open narrative’ portion of the verbal autopsy as a supplement to the closed-ended quantitative surveys, noting that the open narrative provided much more detailed information on cultural attitudes and health service delivery.^2^ The authors suggest that, while the open narrative is unlikely to be helpful in the classification of biomedical causes of death, the additional information on service delivery and community perceptions of illness might be important for evaluating interventions and stimulating accountability for service provision.^2^ Contrary to the author’s contention that the narratives may not help identify the cause of death, we found that specific causes of death were more likely to benefit from the addition of qualitative data than others. While our sample size is small, and we did not specifically ask coders to determine if the additional information provided by the qualitative data enhanced their assessment of the biomedical cause of death, future research with a larger sample size that can ask and answer such a question is warranted.

Other researchers have used word frequency counts, machine learning, and other automated methods to identify the cause of death from the open narrative portions of verbal autopsies.^4,14–16^ Notably, Danso et al.^16^ found that the combination of quantitative and qualitative data yielded the highest sensitivity when examining the cause of death among 6407 verbal autopsies with open narratives of infant deaths in Ghana.^16^ While their study was focused narrowly on identifying the biomedical cause of death, our data suggest the same: quantitative data on its own is valuable, but its value in contributing to our deeper understanding of the factors surrounding deaths can be enhanced with the addition of thoughtfully collected qualitative data.

This research has several important implications for researchers and those seeking to better understand the social and cultural factors that contribute to clinical causes of death in low-resource settings. First, our data indicate that 95% of the time, the VASA-QUAL supplement added meaningful information about the recognition of illness. In settings where illness recognition remains a challenge, including supplemental qualitative modules in VASA studies could assist in generating programmatic recommendations. Second, our data indicate that agreement between quantitative data and qualitative data varies across domains. It would be helpful in future studies with larger sample sizes to explore statistically variability across domains by cause of death. This was not possible in our sample of 48 deaths, but it raises important questions about whether there might be value in following up with families whose children have died from certain causes with lower rates of agreement across qualitative and quantitative data. It also speaks to the value of including qualitative data if and when the verbal autopsy findings are indeterminant or when the physician coders indicate a lack of certainty in their judgement of the cause of death. Finally, when using the Pathway to Survival model to interpret our data, we found that the qualitative supplement was particularly valuable across all causes of death in understanding the choice of outside care during a childhood illness episode. In settings where the choice of care – traditional vs allopathic, local clinic vs hospital – appears to be an important driver of outcomes, additional qualitative explorations may be warranted.

One concern among researchers and programmatic staff has been the feasibility of including a rigorous qualitative component within a large VASA project. While we readily admit that qualitative data collection and analysis are labor intensive and often yield complex findings, our results suggest that certain situations may warrant additional effort. As described, care-seeking pathways are often complicated, especially for children with chronic conditions or conditions that unfold over a longer period than a single, acute event. A qualitative inquiry may be required to understand the care-seeking and care-receiving process truly. It is also possible to consider alternative structures within VASA studies, such as where a subsample of patients is given the supplemental VASA-QUAL at the same time the standard VASA is administered. In other settings, it may make sense to return to families whose children were determined to have died from a given illness to seek additional information through the VASA-QUAL only after the cause of death is known. Such mixed-methods inquiry is no doubt more labor intensive, however, it is likely to yield rich information that may help unpack some of the complexities inherent in childhood deaths that are still occurring within a few kilometers of high-level health centers, such as was the case in our study.

This study has several strengths. First, the structured VASA instrument and the qualitative VASA-QUAL supplement were administered in the same sitting, meaning that respondents were unlikely to suffer memory gaps between data collection episodes. Data were collected by trained local interviewers, with feedback on their interviewing and probing skills provided after the first few interviews. This study also included as close to all families in the catchment area who suffered a U5 loss within the previous 6 months as could be identified, rather than selecting a sub-sample. Finally, this study is the first of which we are aware that explicitly compares data from a quantitative VASA against a qualitative supplement with specific probes, rather than an unspecified ‘open narrative.’

Nonetheless, this study has limitations worthy of discussion. The number of deaths in this catchment area during the study period was relatively small, and deaths may have been missed despite our best efforts to identify each death. While we could have extended the recall period to boost our sample size, we wanted to ensure maximum accuracy of respondents’ memories by limiting recall to 6 months. With such a small sample size, comparisons across different causes of death should be interpreted cautiously. We were unable to run statistical tests, and thus what is reported here reflects frequencies and observations rather than statistically significant findings. Nonetheless, we believe that this study is the first step toward a better understanding of how best to utilize qualitative supplements to standard VASA tools.

## CONCLUSIONS

In conclusion, the use of supplemental qualitative modules can enhance the value of standardized verbal and social autopsy tools in understanding the factors leading up to U5 deaths. Yet, the utility of a qualitative supplement appears to vary both by cause of death and by Pathway to Survival indicator. This raises important questions about resource allocation, given the time and expense associated with adding qualitative modules to an already lengthy VASA interview. Depending upon the research or project goals, the supplemental modules may be best employed when there is a particular interest in understanding symptom recognition or the choice of outside care sought for a childhood illness.

## Figures and Tables

**Table 1.** Demographic Characteristics (N=48)

Variable	N (%)
Gender	
Female	24 (50.0)
Male	24 (50.0)
Age of child at death	
< 6 months	19 (39.5)
6 months - < 1 year	11 (22.9)
1 year - 3 years	14 (29.2)
>3 years	4 (8.3)
Place of Death	
At home	15 (31.3)
At hospital / health center	27 (56.3)
En route	6 (12.5)
Underwent some treatment at home	40 (83.3)
Drugs	38 (95.0)
Herbs	1 (2.5)
Other	1 (2.5)
Sought treatment outside the home	40 (83.3)
Hospital	19 (47.5)
Health Center	10 (25.0)
Private Clinic	11 (27.5)
Sought care at 2+ places	15 (37.5)
Received any treatment at a health facility for the final illness	38 (79.2)

**Table 2.** Agreement between quantitative and qualitative VASA data by indicator

Indicator	Percent agreement between quantitative and qualitative data	Kappa	P value
Recognition of illness	93.75	0.368	0.004
Received, sought, or tried to seek care	91.49	0.308	0.002
Any home care	87.50	0.330	0.011
Choice of outside care	89.58	0.259	0.004
Place of death	81.25	−0.038	0.674

**Table 3.** Examples of additions from the VASAQUAL that were considered meaningful

Recognition of Illness	
Quantitative data: No symptoms observed prior to the child’s death	Qualitative data: Describes baby crying a lot, ‘white things’ in the baby’s nose and mouth, black and odorous stool
Received, sought, or tried to seek care	
Quantitative data: Sought care at hospital for illness leading to death	Qualitative data: Went to the clinic for cough ⟶ treated cough, but it persisted ⟶ returned to clinic ⟶ referred to hospital diagnosed with heart malformation ⟶ went back to hospital every month for checkups ⟶ developed abdominal swelling and bloody stools ⟶ underwent abdominal operation ⟶ died
Any home care	
Quantitative data: Used drugs at home	Qualitative data: Grandmother gave oral rehydration solution at home, as well as deworming medicine and additional tablets provided at the clinic
Choice of outside care	
Quantitative data: No outside care sought	Quantitative data: Mother did not seek outside treatment because she thought the fever would subside after administering paracetamol. She also thought fevers were not unusual in babies and did not foresee a fever being fatal.
Place of death	
Quantitative data: Child died at home.	Qualitative data: Child was found cold but still breathing at home and was rushed to the clinic where she was pronounced dead.
